# Supplementary material for: Self-selection of dissipative assemblies driven by primitive chemical reaction networks
Source: Nat Commun. 2018 May 23;9:2044. doi: 10.1038/s41467-018-04488-y (PMC5966463; doi:10.1038/s41467-018-04488-y)
Supplement: Supplementary file 1 — Supplementary Information [file 41467_2018_4488_MOESM1_ESM.pdf]

Self-selection of dissipative assemblies driven by primitive chemical reaction networks

Tena-Solsona et al.

Supplementary Information

Supplementary Tables

Supplementary Table 1. Characterization of all main products by HPLC

Name	Structure	HPLC retention time	HPLC calibration value (meas.)	HPLC calibration value (calcul.)
**C_3C_3**	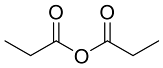	13.9 min (method 1)<br>10.4 min (method 3)	1.9<br>mAU/mM	
**C_3C_4**	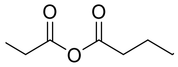	18.8 min (method 2)<br>11.5 min (method 3)		2.1<br>mAU/mM
**C_3C_5**	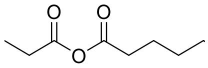	12.4 min (method 3)<br>Coincides with C_4C_4<br>(method 3)		2.3<br>mAU/mM
**C_3C_6**	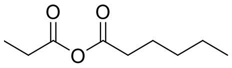	13.1 min (method 3)<br>Coincides with C_5C_4<br>(method 3)		2.4<br>mAU/mM
**C_4C_4**	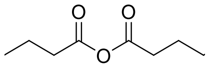	18 min (method 1)<br>12. 4 (method 3)<br>Coincides with C_5C_3<br>(method 3)	2.3<br>mAU/mM	
**C_4C_5**	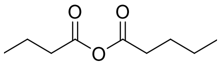	13.1 min (method 3)<br>Coincides with C_6C_3<br>(method 3)		2.4<br>mAU/mM
**C_4C_6**	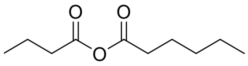	14.0 min (method 3)<br>Coincides with C_5C_5<br>(method 3)		2.5<br>mAU/mM
**C_5C_5**	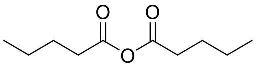	14.0 min (method 3)<br>Coincides with C_4C_6<br>(method 3)	2.5<br>mAU/mM	
**C_5C_6**	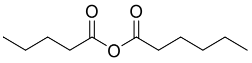	14.4 min (method 3)		2.6<br>mAU/mM
**C_6C_6**	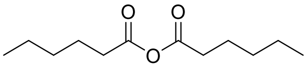	24.3 min (method 2)<br>14.8 min (method 3)	2.6<br>mAU/mM	

Supplementary Table 2. Characterization of all side products by HPLC and ESI-MS

Name	Structure	Mass calculated [g/mol]	Mass found [g/mol]	HPLC retention time*	HPLC calibration value (meas.)
**C_3 N-acylurea**	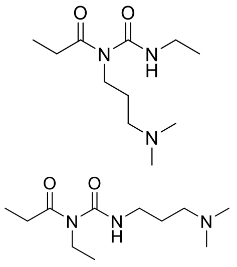	Mw = 229.32<br>$C_{11}H_{23}N_3O_2$	230.15<br>$[Mw+H]^+$	9.0 min<br>(method 1)	104 mAU/mM
**C_4 N-acylurea**	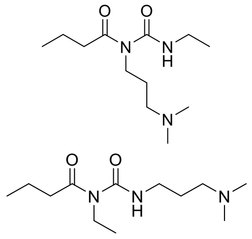	Mw = 243.35<br>$C_{12}H_{25}N_3O_2$	244.15<br>$[Mw+H]^+$	10.5; 10.7 min<br>(method 2)	109 mAU/mM
**C_5 N-acylurea**	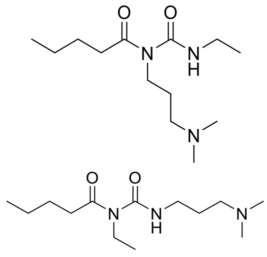	Mw = 257.38<br>$C_{13}H_{27}N_3O_2$	258.26<br>$[Mw+H]^+$	8.7; 8.9 min<br>(method 3)	136 mAU/mM
**C_6 N-acylurea**	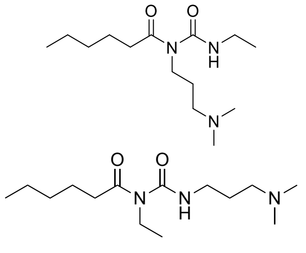	Mw = 271,41<br>$C_{14}H_{29}N_3O_2$	272.24<br>$[Mw+H]^+$	13.4; 13.8 min<br>(method 2)	138 mAU/mM

Supplementary Table 3. k-values for all the reactions describe in our kinetic model

k-values for C_3, C_4, C_5 and C_6 experiments when not competing

Precursor	*Range of EDC (mM)*	*[acid] (M)*	**k_1** (M^{-1} s^{-1})	**k_2** (M s^{-1})	**k_3** (s^{-1})	**k_4** (s^{-1})	**k_5** (s^{-1})	*Solubility of anhydride (mM)*
C_3	*2-50*	*0.3*	$2.3*10^{-2}$	$10*k_1$	$3.9*k_1$	$1.5*10^{-2}$	$1.6*10^{-3}$	*N.A.*
C_4	*2-50*	*0.3*	$2.3*10^{-2}$	$10*k_1$	$2.3*k_1$	$1.5*10^{-2}$	$1.1*10^{-3}$	*11*
C_5	*2-10*	*0.3*	$2.3*10^{-2}$	$10*k_1$	$2.3*k_1$	$0.9*10^{-2}$	$1.1*10^{-3}$	*1.0*
C_6	*2-10*	*0.1*	$2.3*10^{-2}$	$10*k_1$	$1.2*k_1$	$0.9*10^{-2}$	$1.1*10^{-3}$	*0.05*

k-values for the competition experiments between C_3 and C_5

Precursor	*Flux of EDC (mM hr^{-1})*	*[acid] (M)*	**k_1** (M^{-1} s^{-1})	**k_2** (M^{-1} s^{-1})	**k_3** (s^{-1})	**k_4** (s^{-1})	**k_5** (s^{-1})	*Solubility of anhydride (mM)*
C_3	*5-35*	*0.3*	$2.3*10^{-2}$	To form C_3C_3: $10*k_1$	$3.0*k_1$	$1.5*10^{-2}$	Hydrolysis of C_3C_3 $2.5*10^{-3}$	C_3C_3 *N.A.*
C_3		*0.3*		To form C_3C_5: $10*k_1$			Hydrolysis of C_3C_5 $2.0*10^{-3}$	C_3C_5 *N.A.*
C_5	*5-35*	*0.3*	$2.3*10^{-2}$	To form C_5C_5: $10*k_1$	$0.7*k_1$	$1.5*10^{-2}$	Hydrolysis of C_5C_5 $1.8*10^{-3}$	C_5C_5 *0.8*
C_5		*0.3*		To form C_3C_5: $10*k_1$				

k-values for the competition experiments between C_3 and C_6

Precursor	*Flux of EDC (mM hr^{-1})*	*[acid] (M)*	**k_1** (M^{-1} s^{-1})	**k_2** (M^{-1} s^{-1})	**k_3** (s^{-1})	**k_4** (s^{-1})	**k_5** (s^{-1})	*Solubility of anhydride (mM)*
C_3	*5*	*0.1*	$2.3*10^{-2}$	To form C_3C_3: $10*k_1$	$3.0*k_1$	$1.5*10^{-2}$	Hydrolysis of C_3C_3 $1.6*10^{-3}$	C_3C_3 *N.A.*
C_3				To form C_3C_6: $10*k_1$			Hydrolysis of C_3C_6 $2.0*10^{-3}$	C_3C_6 *N.A.*
C_6	*5*	*0.1*	$2.3*10^{-2}$	To form C_6C_6: $10*k_1$	$0.4*k_1$	$0.9*10^{-2}$	Hydrolysis of C_6C_6 $2.0*10^{-3}$	C_6C_6 *0.04*
C_6				To form C_3C_6: $10*k_1$				

Supplementary Figures

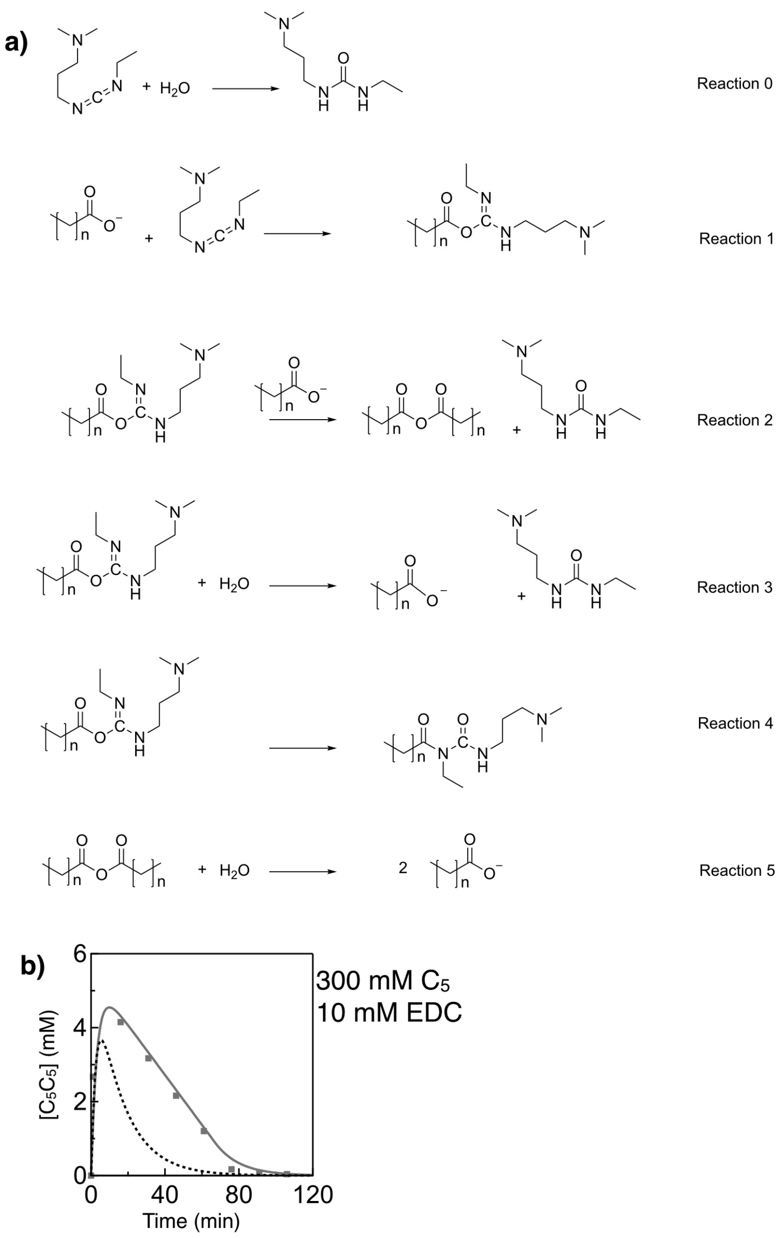

Supplementary Figure 1. Chemical reactions taken into account in the kinetic model. a) Reaction 0 represents the direct hydrolysis of EDC carbodiimide. Reaction 1 corresponds to the formation of O-acyl urea by reaction with EDC. Reaction 2 corresponds to the formation of the anhydride. Reaction 3 shows the direct hydrolysis of the O-acyl urea. Reaction 4 corresponds to the formation of the unreactive N-acyl urea. Reaction 5 shows the hydrolysis of the anhydride. **b**) HPLC (markers) and model (lines) data of the concentration of anhydride C_5C_5 when 300mM C_5 is fueled with 10 mM EDC. The solid black line represents model data with the inhibition mechanism. The dashed black line represents model data that does not take into account the inhibition mechanism inhibition.

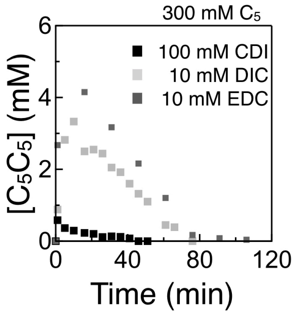

Supplementary Figure 2. Anhydride concentration over time in response to various fuels. The concentration of C_5C_5 when 300 mM C_5 was subjected to 100 mM CDI, 10 mM DIC or 10 EDC mM.

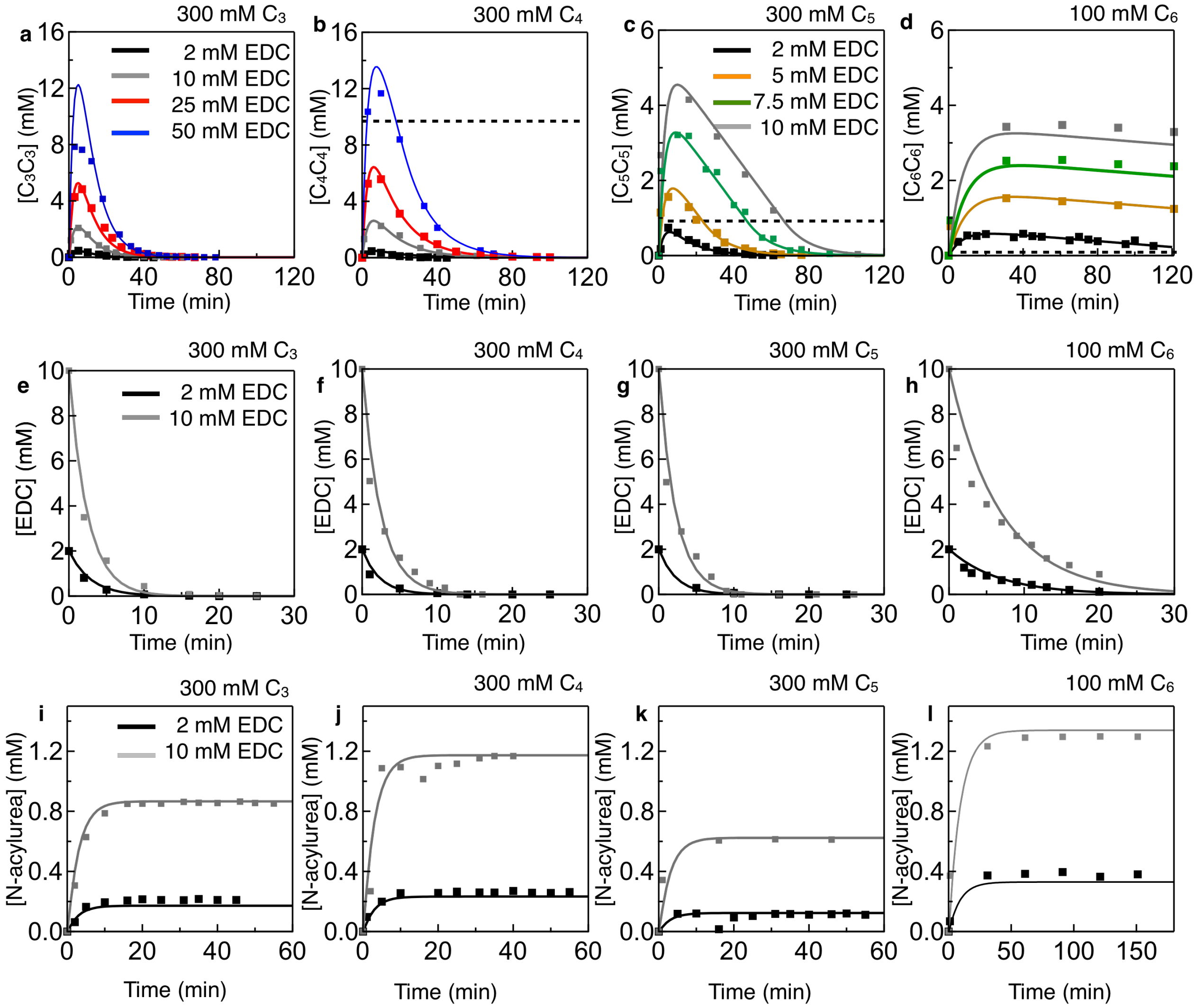

Supplementary Figure 3. HPLC and model data. HPLC (markers) and model (lines) data of the concentration of anhydride (**a-d**), the concentration of EDC (**e-f**) and the concentration of the corresponding N-acyl urea (**i-l**) for 300 mM C_3, C_4, C_5 or 100 mM C_6 in response to various concentrations of EDC. The dash horizontal lines equal the solubility of C_4C_4 (**b**), C_5C_5 (**c**) and C_6C_6 (**d**).

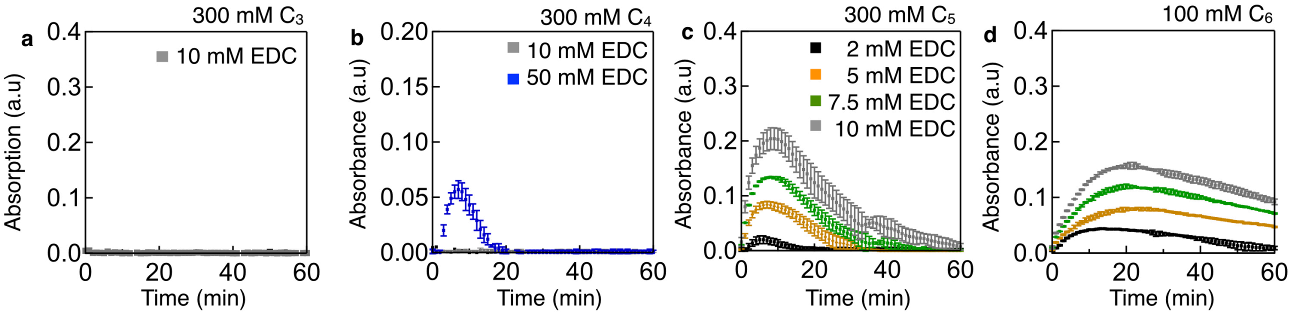

Supplementary Figure 4. UV/Vis data of precursors in response to fuel in non-competition experiments. Absorbance of 600 nm light as a measure of turbidity against time for 300 mM C_3 in response to 10 mM EDC, **b**) for 300 mM C_4 in response to 10 and 50 mM EDC, **c**) for 300 mM C_5 in response to 2, 5, 7.5 and 10 mM of EDC and **d**) 100 mM C_6 in response to 2, 5, 7.5 and 10 mM of EDC. Error bars depict the standard deviation of three experiments.

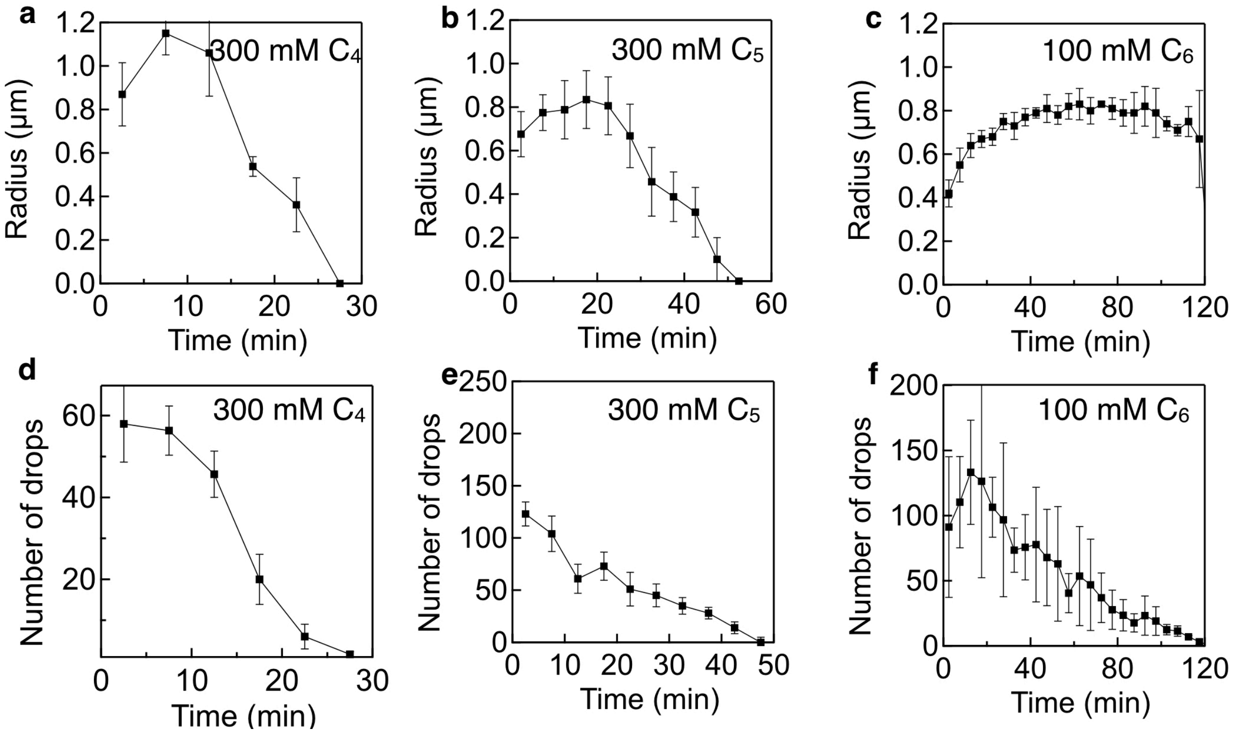

Supplementary Figure 5. Analysis of confocal microscopy data in non-competitions experiments. a-c) Average radius and **d-f**) number of droplets in the micrographs when 300 mM C_4 was fuelled with 50 mM EDC, 300 mM C_5 was fuelled with 10 mM EDC or 100 mM C_6 was fuelled with 2 mM EDC. Error bars depict the standard deviation of three experiments, lines are added to guide the eye.

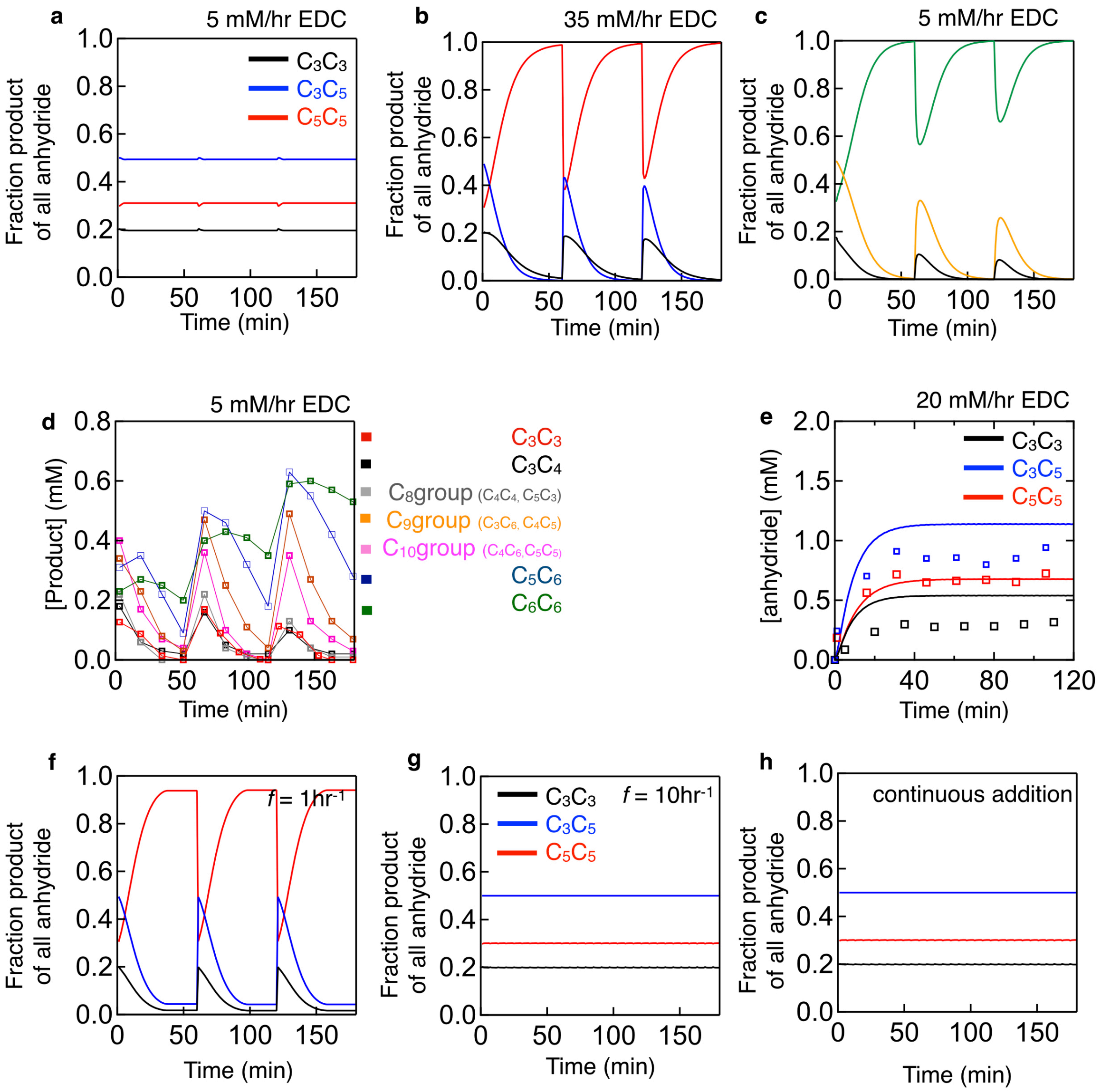

Supplementary Figure 6. Supporting data for competition experiments. a-c, Plots of the fraction of product compared to all anhydride over time when a mixture of 300 mM C_3 and 300 C_5 is fuelled with 5 mM (**a**) or 35 mM (**b**) EDC every hour or when a mixture of 100 mM C_3 and 100 C_6 is fuelled with 5 mM EDC every hour (**c**). **d**, Concentration anhydride against time when 100 mM C_3, 100 mM C_4, 100 mM C_5 and 100 C_6 are competing for 5 mM EDC every hour. Makers correspond to the measured HPLC data, the lines are added to guide the eye. **e**, Concentration anhydride against time when a mixture of 300 mM C_3 and 300 C_5 is continuously fuelled EDC with a microsyringe pump to a flux of 20 mM hr^{-1}. Makers correspond to the measured HPLC data, whereas the lines correspond to the calculated data. The calculated data deviates somewhat from the HPLC data, likely a result of the fact that the experiment is stirred, while the experimental data used to fit the model was acquired from samples that were not stirred. **f-h**) Plots of the fraction of product of all anhydride over time when 300 mM C_3 and 300 C_5 is fuelled with 60 mM EDC over a three-hour experiment. The fuel is either delivered once per hour in 20 mM batches (1hr^{-1}, **f**), 30 batches or 10 times per hour in 2 mM batches (10 hr^{-1}, **g**) or continuously (**h**).

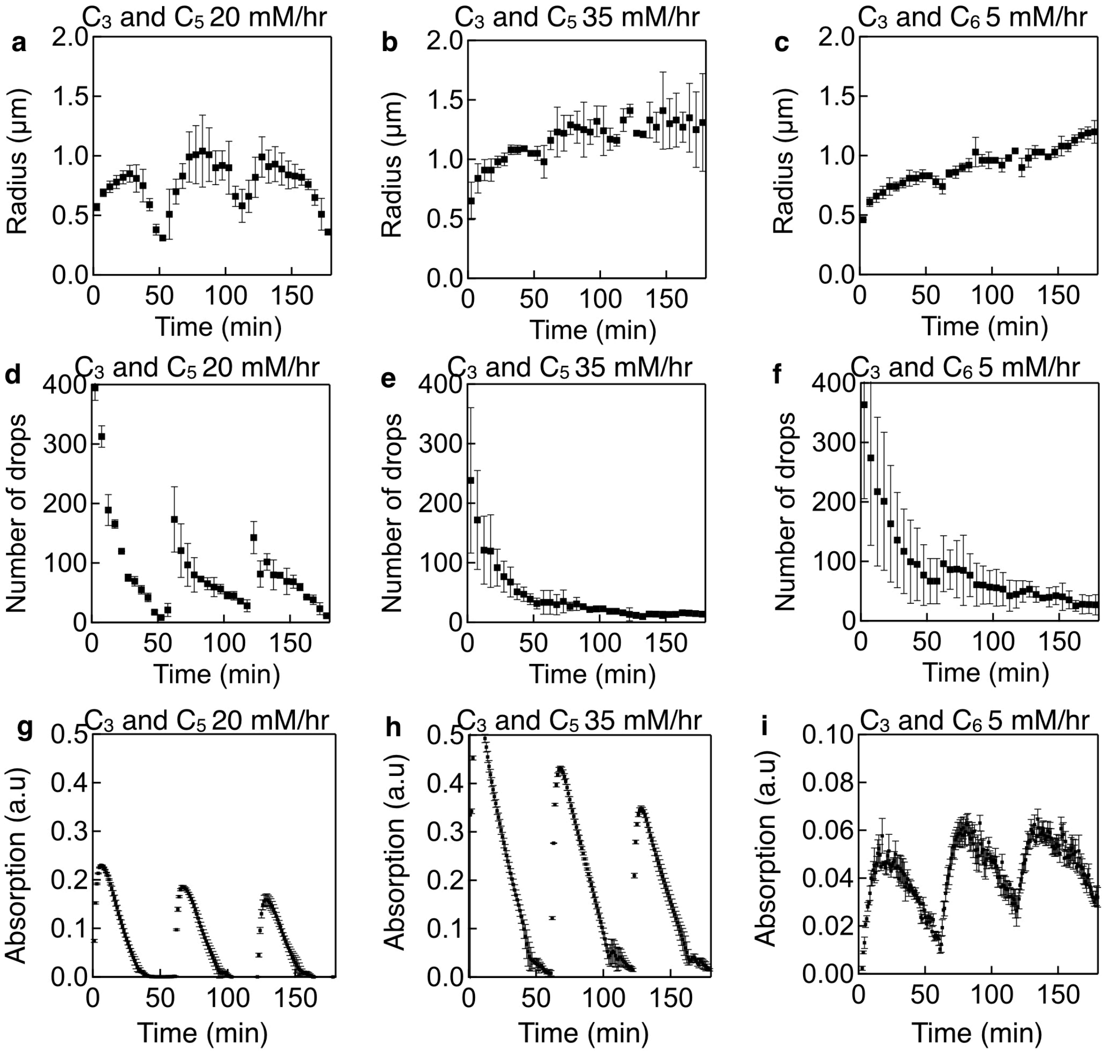

Supplementary Figure 7. UV/Vis data and confocal microscopy data response to fuel in competition experiments. a-c, Average radius of the droplets when 300 mM C_3 and 300 mM C_5 was fuelled with 20 mM (**a**) or with 35 mM (**b**) every hour, or when 100 mM C_3 and 100 mM C_6 was fuelled with 5 mM every hour (**c**). **d-f,** Average number of droplets found in each micrograph under the condition described in **a**, **b** and **c**, respectively. Every minute the solution was imaged. Data of five minutes was binned for statistical analysis. Error bars refer to the standard deviation between experiments (n=3). **g-i**, Absorbance of 600 nm light as a measure of turbidity against time for the experimental condition described in a-c, respectively. Note that both the 20 mM trace drops to zero before addition of new fuel whereas the 35 and 5 mM hr^{-1} did not. Error bars depict the standard deviation of three experiments.
